# Highly resolved taphonomic variations within the Early Ordovician Fezouata Biota

**DOI:** 10.1038/s41598-024-71622-w

**Published:** 2024-09-06

**Authors:** Farid Saleh, Jonathan B. Antcliffe, Enzo Birolini, Yves Candela, Nora Corthésy, Allison C. Daley, Christophe Dupichaud, Corentin Gibert, Pauline Guenser, Lukáš Laibl, Bertrand Lefebvre, Soline Michel, Gaëtan J.-M. Potin

**Affiliations:** 1 Institute of Earth Sciences, University of Lausanne, Géopolis, 1015 Lausanne, Switzerland; 2 Université Claude Bernard Lyon 1, École Normale Supérieure de Lyon, CNRS, UMR5276 LGL‑TPE, Villeurbanne, France; 3 Department of Natural Sciences, National Museums Scotland, Edinburgh, EH1 1JF UK; 4 Spatial Ecology and Paleontology Laboratory (SEPL), School of Biological Sciences, Georgia Institute of Technology, Atlanta, GA USA; 5 Czech Academy of Sciences, Institute of Geology, Rozvojová 269, 165 00 Prague 6, Czech Republic

**Keywords:** Statistics, Invertebrates, Early Palaeozoic, Gondwana, Palaeontology, Palaeoecology

## Abstract

The Fezouata Biota (Morocco) is a Burgess Shale-type (BST) assemblage that provides a wealth of information on Early Ordovician ecosystems. Much work has been done to compare the preservation of the Fezouata Biota to other BSTs. However, studies investigating preservation variations within the Fezouata Biota are rare. Here, we use probabilities to investigate the preservation of various ecological categories of Fezouata eumetazoans. Complex taphonomic processes and phylum-specific constraints have led to the better preservation of predators/scavengers in this biota. However, no differences in preservation are observed between vagile and sessile taxa. Importantly, Tremadocian taxa are better preserved than Floian ones. As such, this study highlights the gradual closure of the BST window of preservation in the Zagora region of Morocco and constitutes a benchmark for future palaeoecological and evolutionary studies on the Fezouata Biota.

## Introduction

**Fig. 1:**
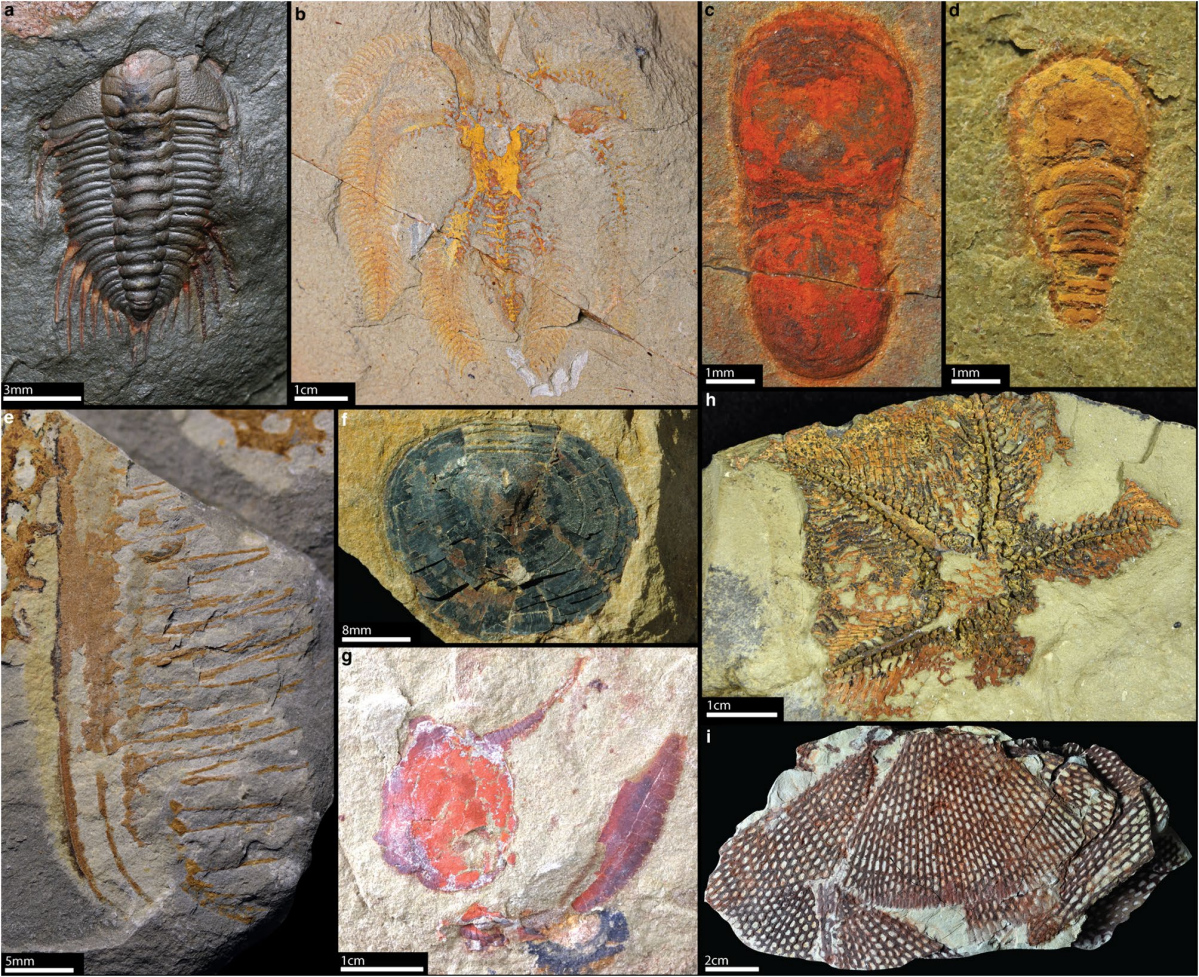
Selected animals from the Early Ordovician Fezouata Biota and the contextualization of the current work. Arthropods (**a**–**e**): (**a**) trilobite *Anacheirurus* YPM.530933, (**b**) marrellid MGL.102397, (**c**) nektaspidid *Tariccoia tazagurtensis* MGL.102155a, (**d**) chelicerate *Setapedites abundantis* MGL.102872, and (**e**) frontal appendage of radiodont *Pseudoangustidontus izdigua* MGL.103606. (**f**) The brachiopod *Orbithele* UCBL.FSL711960. Echinoderms (**g**, **h**): (**g**) *Nimchacystis agterbosi* UCBL.FSL713120; (**h**) *Villebrunaster fezouataensis* UCBL.FSL424961. (**i**) The graptolite *Sagenograptus murrayi* AA.BIZ3.OI.35. AA: Cadi-Ayyad University (Morocco); MGL: Musée de Géologie de Lausanne (Switzerland); UCBL: Université Claude Bernard (France); and YPM: Yale Peabody Museum (USA).

The Early Ordovician Fezouata Biota offers unparalleled views into marine ecosystems over 470 million years ago. Discovered in Morocco's Draa Valley, this fossil treasure trove boasts an extraordinary diversity of well-preserved organisms, from soft-bodied animals to fossilized biomineralized organisms. The Fezouata Biota contains animals such as annelids^1^; arthropods^2–19^; brachiopods^20^; echinoderms^21–28^; hemichordates^29,30^; hyoliths^31,32^; molluscs^33–37^; scalidophorans^38^, among other taxa^39–43^ (Fig. 1a–i). Two fossiliferous sedimentary intervals have yielded the most exceptionally preserved Fezouata Biota fossils. The first is late Tremadocian in age^45–48^, and the second dates from the middle to late Floian^49^. The Fezouata Biota environment was dominated by wave/processes and modulated by tides^50–52^. However, most exceptionally preserved animals were preserved in distal settings below the storm-weather wave base^52^.

Fossils from the Fezouata Biota show that animal groups characteristic of Cambrian Burgess Shale-type (BST) communities, such as radiodonts, lived into the Early Ordovician, highlighting a gradual transition between the Cambrian and the Ordovician^44,45^. When they were originally discovered, fossils from the Fezouata Biota also indicated that the BST mode of preservation was operational at least until the Early Ordovician. In fact, the Fezouata Biota fossils share many preservational similarities with Cambrian BST communities. The primary mode of preservation of Fezouata Biota exceptionally preserved fossils is carbonaceous compressions with accessory pyritization^52–55^. However, most carbon was leached due to weathering processes and replaced by iron oxides, giving the fossils their red colour^39,52^ (Fig. 1a–i).

The Fezouata Biota provided, and continues to provide, a wealth of information on the ecology of ancient ecosystems. However, as in other BSTs, taphonomic constraints must be accounted for to extract robust ecological information^56^. Ecology and taphonomy are connected in Lagerstätten, as the ecological context of an organism profoundly influences its preservation potential in the fossil record. Probabilistic methods have proven to be a valuable tool to investigate how ecology influences preservation in Lagerstätten (Fig. 1j). Using such methods, the Fezouata Biota was shown to differ significantly from other Cambrian BSTs, because entirely soft non-cuticularized organisms are lacking and because internal soft cellular structures are mostly preserved in association with a sclerotized or partially mineralized external surface of the body^56^. It was however shown that the Fezouata Biota equally preserves epibenthic, nektobenthic, and nektonic communities^57,58^. Yet, detailed taphonomic investigations on the preservation potential of other ecological and evolutionary traits, such as feeding strategies, motility, and age categories, remain unexplored and many open questions persist: Are sessile taxa better preserved than vagile ones? Are suspension feeders better captured than predators? Are there variations in preservation between Tremadocian and Floian taxa?

Here, we answer these questions by highlighting that significant preservation variations exist within all categories (except motility). This is likely due to a complex interplay between preservation mechanisms and phylum-specific constraints.

## Material and methods

Probabilistic methodological details have been previously published^56–60^. The dataset consists of a list of preserved eumetazoan genera in the Fezouata Biota (see Supplementary Material), together with information on the preserved biological structures in each genus. Biological structures were divided into five types of tissues (noted A, B, C, D, and E) based on their resistance to the decay process in general (Table 1). The five tissue types describe the original anatomical composition of organisms rather than the result of a specific taphonomic pathway. For example, a pyritized digestive system would be classified under “E”, which encompasses internal organs, rather than “A”, which is for biomineralized structures. To determine the occurrences of tissues for each genus, the best-preserved specimen of the genus or a combination of specimens is taken into account. For instance, if there are 99 fragmentary samples and only one complete specimen for a certain genus, the complete specimen is used to fill in the database. If another genus has two specimens, one shows a biomineralized structure and a gut, while the other preserves a non-sclerotized cuticle, this genus is considered to preserve biominerals, non-sclerotized cuticles, and internal organs.

**Table 1:** Preservation data tabulated for Fezouata Biota genera.

Biological structures	Defintions	Examples
A	Biomineralised structures, regardless of the nature or degree of mineralization	Trilobite and echinoderm exoskeletal elements
B	Sclerotized structures that are hard or plate-like	Rhabdosome/tubarium of graptolites and the carapaces of many bivalved arthropods
C	Non-sclerotized cuticularized bodies formed of polysaccharides	Body walls of annelids and parts of non-biomineralized arthropods (e.g., arthrodial membranes)
D	Composed solely of cells and are in direct contact with the surrounding environment	Tentacles of cnidarians, brachiopods, and hyoliths
E	Internal organs and systems	Digestive, respiratory, and nervous systems

Note that any given genus can preserve one or more types of tissues concurrently.

Genera were then divided into three different categories and seven subcategories according to their ecological characteristics (Table 2). Classifying genera within these categories and subcategories is based on the adult morphology of each genus, whenever known. For example, if a genus has motile larvae and sessile adults, it is coded as sessile in the database. A certain overlap exists between the ecological categories (Table 2). In theory, six ecological combinations exist (e.g., sessile predators/scavengers; sessile suspension feeders; sessile organisms that are neither predators nor suspension feeders; vagile predators/scavengers; vagile suspension feeders; vagile organisms that are neither predators nor suspension feeders). In the Fezouata Biota, all sessile animals are suspension feeders. Nonetheless, this does not imply that all suspension feeders are sessile.

**Table 2:** Ecological characteristics tabulated for Fezouata Biota genera.

Categories	Definitions	Subcategories	Definitions	Examples
Motility	The quality of being able to move or not	Sessile	Not free to move	brachiopods, bivalves
		Vagile	Move on the seafloor or free swimmers	annelids, cephalopods, panarthropods
Feeding strategy	The type of behaviour best suited to gather food	Predators/scavengers	Actively hunt and kill prey; or feed on carrion	chelicerates; annelids
		Suspension feeders	Capture food particles suspended in water	echinoderms, brachiopods
		Other strategies	Grazing among others	some gastropods
Age	The age of sedimentary layers where taxa are found	Strictly Tremadocian	Found only in Tremadocian layers	*Procothurnocystis*
		Strictly Floian	Found only in Floian layers	*Milonicystis*

These include motility, feeding strategy, and age. The examples presented are not exclusive, and other animal groups could belong or have at least some of their genera in each of the below categories (detailed examples are given in the Supplementary Material).

All previous ecological categories have representatives in both Floian and Tremadocian intervals. Note that taxa present in both the Tremadocian and Floian were not accounted for when comparing the preservation potential of these intervals to avoid any overlap between them (Table 2). In other words, including a category with taxa present in both the Floian and Tremadocian intervals when investigating preservation differences between these intervals is misleading because a sedimentary layer cannot be both Tremadocian and Floian at the same time. For taxa present in both Tremadocian and Floian intervals, we have information on the best-preserved specimen overall, without knowing the preservation state of other representatives of this taxon in the other interval. This information would be necessary to understand preservational differences between the Floian and Tremadocian for taxa present in both intervals. However, all taxa including those present in both Floian and Tremadocian intervals were considered when comparing the preservation potential of various ecological categories (i.e., differences between motilities and feeding strategies; Table 2 and Supplementary Material).

The proportions of different tissue occurrences and co-occurrences within each subcategory (Table 2) were plotted and numerous preservational indices were then calculated. For a certain subcategory in Table 2, probabilities of genera with one [P(1s); i.e., A, or B, or C, or D, or E], two [P(2s); e.g., AB], three [P(3s); e.g., ABC], or four [P(4s); e.g., ABCE] tissues in addition to the probabilities of biomineralized P(A); sclerotized P(B); cuticularized P(C); cellular structures in contact with water P(D); and internal organs P(E) were calculated. The average number of tissues per taxon was also calculated. Note that most of the obtained values could be impacted by anatomical heterogeneities between taxa (i.e., not all genera have the same type of tissues in life). For example, a trilobite does not have cellular sheets in contact with the seawater. This is not true for P(E) because all eumetazoans have internal organs. As such, P(E) is considered the baseline to understand preservation variations.

To investigate how the different phyla influence the calculated indices, the proportions of phyla in each subcategory (Table 2) were plotted. Then, to examine whether preservation variations could be observed within a certain phylum, the aforementioned indices were recomputed for all subcategories, based solely on panarthropod data rather than the complete dataset. Finally, to determine whether the probabilities of internal organ preservation between subcategories show significant differences, a binomial model was used, following the methodology of^56–60^. Raw data are provided in the Supplementary Material file.

## Results and discussion

**Fig. 2:**
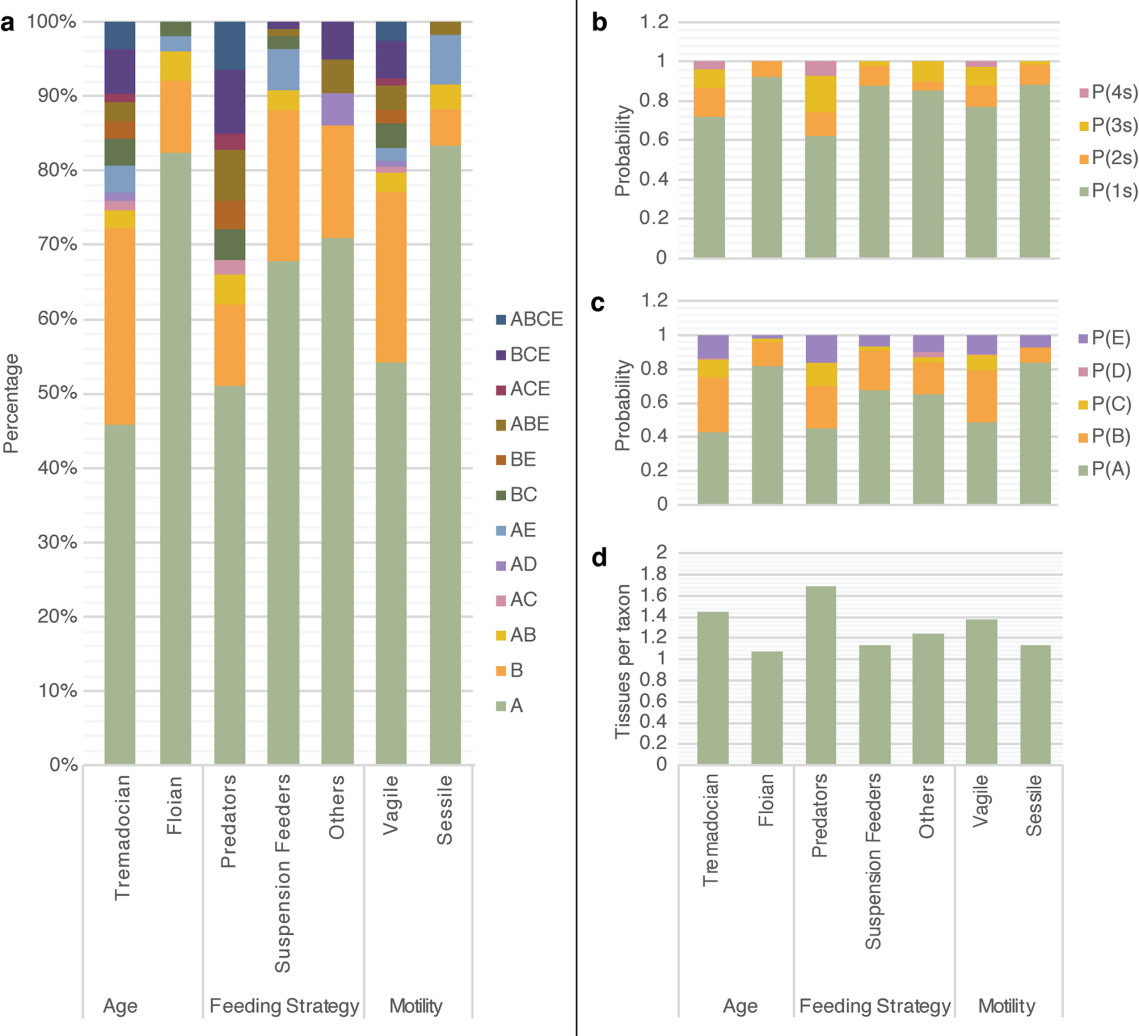
Probabilistic framework for understanding preservation in the Fezouata Biota. (**a**) Diversity of tissue type associations within various age, feeding, and motility categories. The different tissues: “A” biomineralized; “B” sclerotized; “C” cuticularized; “D” cellular sheets in contact with the water column; and “E” internal organs. (**b**) Probabilities of taxa preserving one (1 s), two (2 s), three (3 s), and four (4 s) tissue types. (**c**) Probabilities of preserved tissue types [P(E); P(B); P(C); P(D) and P(E)]. (**d**) Average number of tissue types per taxon.

In the Fezouata Biota, Tremadocian organisms, predators/scavengers and vagile genera show the highest diversity of tissue-assemblage associations (Table 3, Fig. 2a). These are also the only subcategories preserving associations of four tissue types (Fig. 2b). Internal organs are the most prevalent in Tremadocian, predators/scavengers and vagile taxa (Fig. 2c). The average number of tissues per taxon (Fig. 2d) shows more or less a similar pattern to the probability of internal organs (Fig. 2c). In summary, it is possible to differentiate the preservation of Tremadocian versus Floian animals (Fig. 2a,b,c), predators/scavengers and non-predators/non-scavengers (Fig. 2a,b,d), and vagile versus sessile taxa (Fig. 2a,c,d).

**Table 3:** Summary statistics of the raw data.

	Age		Feeding strategy			Motility	
	Tremadocian	Floian	Predators/scavengers	Suspension feeders	Other	Vagile	Sessile
Number of taxa	83	51	48	109	21	118	60
A	38	42	24	74	15	64	50
B	22	5	5	22	3	27	3
AB	2	2	2	3	0	3	2
AC	1	0	1	0	0	1	0
AD	1	0	0	0	1	1	0
AE	3	1	0	6	0	2	4
BC	3	1	2	2	0	4	0
BE	2	0	2	0	0	2	0
ABE	2	0	3	1	1	4	1
ACE	1	0	1	0	0	1	0
BCE	5	0	4	1	1	6	0
ABCE	3	0	3	0	0	3	0
1 s	60	47	29	96	18	91	53
2 s	12	4	7	11	1	13	6
3 s	8	0	8	2	2	11	1
4 s	3	0	3	0	0	3	0

The different tissues: “A” biomineralized; “B” sclerotized; “C” cuticularized; “D” cellular sheets in contact with the water column; and “E” internal organs. Note that tissue associations not present in any category (e.g., BD) were removed from the table. Additionally, the total number of taxa in the age category is lower than in the feeding strategy and motility categories because taxa present in both Tremadocian and Floian layers were excluded from the analyses. The raw data showing the taxa belonging to each age, feeding strategy and motility categories are provided in the Supplementary Material.

The difference in the proportions of internal organs between strictly Tremadoian and strictly Floian taxa is significant. It is not possible to replicate P(E) value of Tremadocian taxa using the Floian dataset (i.e., binomial distribution *p-value* = 0.000238077). It was also not possible to replicate the P(E) value of predators/scavengers using the dataset of other feeding strategies (i.e., binomial distribution *p-value* = 0.000000011). However, no significant difference could be observed between vagile and sessile taxa. It was possible to replicate the P(E) value of vagile taxa using the dataset of sessile genera (i.e., binomial distribution *p-value* = 0.088182652). This essentially means that, within their respective categories, Tremadocian taxa and predators/scavengers are better preserved than other subcategories, while no differences in preservation could be observed between sessile and vagile taxa.

**Fig. 3:**
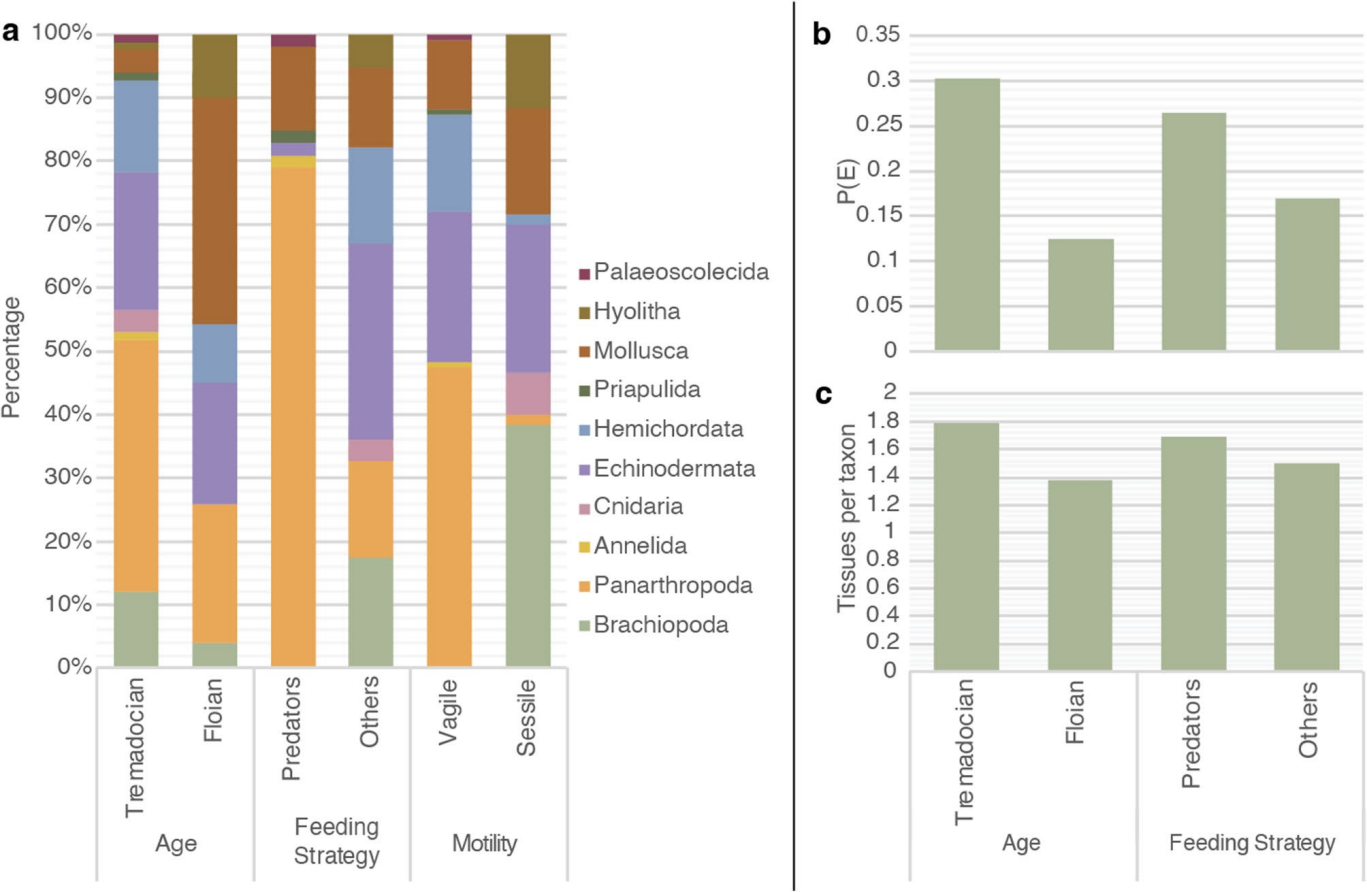
Further probabilistic information on preservation within the Fezouata Biota. (**a**) Distribution of phyla within various age, feeding, and motility categories. (**b**) Variations in the probability of panarthropods' internal organs P(E). (**c**) Variations in the average number of tissues per taxon in panarthropods. Note that a correlation exists between (**b**) and (**c**), although variations are more clearly visible in (**b**).

The non-significant differences between vagile and sessile taxa make sense in the context of the Fezouata Biota. In the Fezouata Biota, most animals were dead and decaying prior to their burial by storm-induced deposits^61^ and a carcass of a motile animal does not behave differently from that of a sessile organism. To explore taphonomic differences within other categories (i.e., age and feeding strategy), the proportions of phyla for each subcategory are plotted (Fig. 3a).

The major difference between Tremadocian and Floian taxa is the high panarthropod and hemichordate diversity, and the low diversity of molluscs in the Tremadocian (Fig. 3a). These observations could explain why Tremadocian taxa are better preserved than Floian ones (i.e., high internal organ probability and high average number of tissues per taxon in the Tremadocian; Fig. 2c,d). It has been previously shown that soft tissues in direct contact with the water column, such as those of molluscs and the tentacles of hyoliths, are the fastest to decay in the Fezouata Biota^56,57^ because they are exposed to internal and external bacteria and scavengers following death, often leading to their rapid loss prior to the stabilization of the carcass. In contrast, rigid body walls formed of mineralized and sclerotized material, fully encapsulating soft internal tissues such as those of panarthropods like trilobites, are key for exceptional fossil preservation in the Fezouata Biota, as they often lead to reducing internal conditions during decay^56^. These reducing conditions are favourable for the stabilization of internal structures through the precipitation of minerals like pyrite^56^. This could be a reason why panarthropods in general show high P(E) values in the Fezouata Biota^56^. Note that these processes are mainly operational in the Fezouata Biota, where preservation consists of dead organisms that suffered from decay before their stabilization. Sites such as the Chengjiang Biota and the Walcott Quarry have different processes than those observed here, as obrution events in these sites managed to capture living taxa^56–58^. For instance, in the Chengjiang Biota, preservation potential increased between nektonic animals such as radiodonts that could avoid obrution events and endobenthic taxa that are easily captured by event flows and sediments^58^. This is not the case in the Fezouata Biota, where all modes of life (i.e., endobiotic, epibenthic, nektobenthic and nektonic) are equally preserved^57^.

The higher preservation potential of panarthropods and the lower preservation potential of animals like molluscs and brachiopods in the Fezouata Biota means that any subcategory with a considerable amount of arthropods and a minimal amount of molluscs and brachiopods tends to show a higher preservation potential than other subcategories. This could explain why predators/scavengers, which are mainly arthropods in the Fezouata Biota (Fig. 3a), are better preserved than other feeding strategies that have a considerable proportion of brachiopods and echinoderms (Fig. 2c,d). Echinoderms decay and disarticulate very rapidly after death. This rapid degradation and disarticulation of echinoderms is well documented in the Fezouata Biota^61^. For instance, exceptional preservation of echinoderms in the Fezouata Biota is observed only in a 1 cm-thick sedimentary layer, which has yielded around 300 specimens of stylophorans in total^61^. Among these hundreds of specimens, around 10 show soft-tissue remains, and only one provides a more or less complete view of the soft tissues. In other words, among the thousands of echinoderm specimens in the Fezouata Biota, only one managed to escape pronounced decay, likely because this organism had not been dead much earlier than the arrival of the burial event^61^.

Considering that the aforementioned phylum-specific anatomical constraints dictate the preservation potential of ecological subcategories, one would expect a significant difference in the preservation of vagile taxa, which are dominated by panarthropods, and sessile taxa, which are dominated by brachiopods (Fig. 3a). However, as indicated earlier, this is not the case as no significant difference is observed between both subcategories (i.e., binomial distribution *p-value* > 0.05). In other words, there must be other taphonomic explanations related to the feeding strategy, and age of different taxa, regardless of whether these taxa are panarthropods, brachiopods, or echinoderms.

To understand the control of feeding strategy and age on exceptional fossil preservation, we focused on the variations in the probability of internal organ preservation P(E), and the average number of tissues per taxon in panarthropods (Fig. 3b,c). Within panarthropods, Tremadocian taxa preserve more tissues per taxon than Floian genera, and predators preserve more tissues per taxon than other feeding strategies (Fig. 3c). A similar pattern is observed for P(E) (Fig. 3b). However, significant differences in P(E) are only observed between Tremadocian and Floian genera. It was not possible to replicate Tremadocian P(E) values using the Floian dataset (binomial distribution *p-value* = 0.018851802). A non-significant difference is calculated for predators/scavengers versus non-predatory/non-scavenging panarthropods, as it was possible to replicate the P(E) value of predators/scavengers using the non-predators/non-scavenging dataset (binomial distribution *p-value* = 0.222745916). This essentially means that the temporal distribution of the taxon controls its preservation within the Fezouata Biota with Tremadocian taxa being better preserved than Floian ones.

The higher preservation potential of Tremadocian taxa could reflect that Tremadocian levels have been more extensively sampled and studied than Floian ones^1–49^. However, there is also a shallowing trend observed between the Tremadocian and the Floian in the Zagora region of Morocco, where the Fezouata Biota is found^50–52^. Additionally, the facies in which most Fezouata Biota fossils are found become rarer in the Floian^48^. In other words, the patterns observed here for the Fezouata Biota suggest that favourable environmental conditions for exceptional fossil preservation were more prevalent in the Tremadocian compared to the Floian, which could also mean the gradual closure of the BST window of preservation in the Zagora region in Morocco. This suggestion does not imply the complete closure of this window in the Ordovician, as exceptional fossil preservation continues to occur in other places late in the Early Ordovician^62^, and animals characteristic of BST communities continue to be discovered later in the Ordovician^63,64^.

## Conclusion

**Fig. 4:**
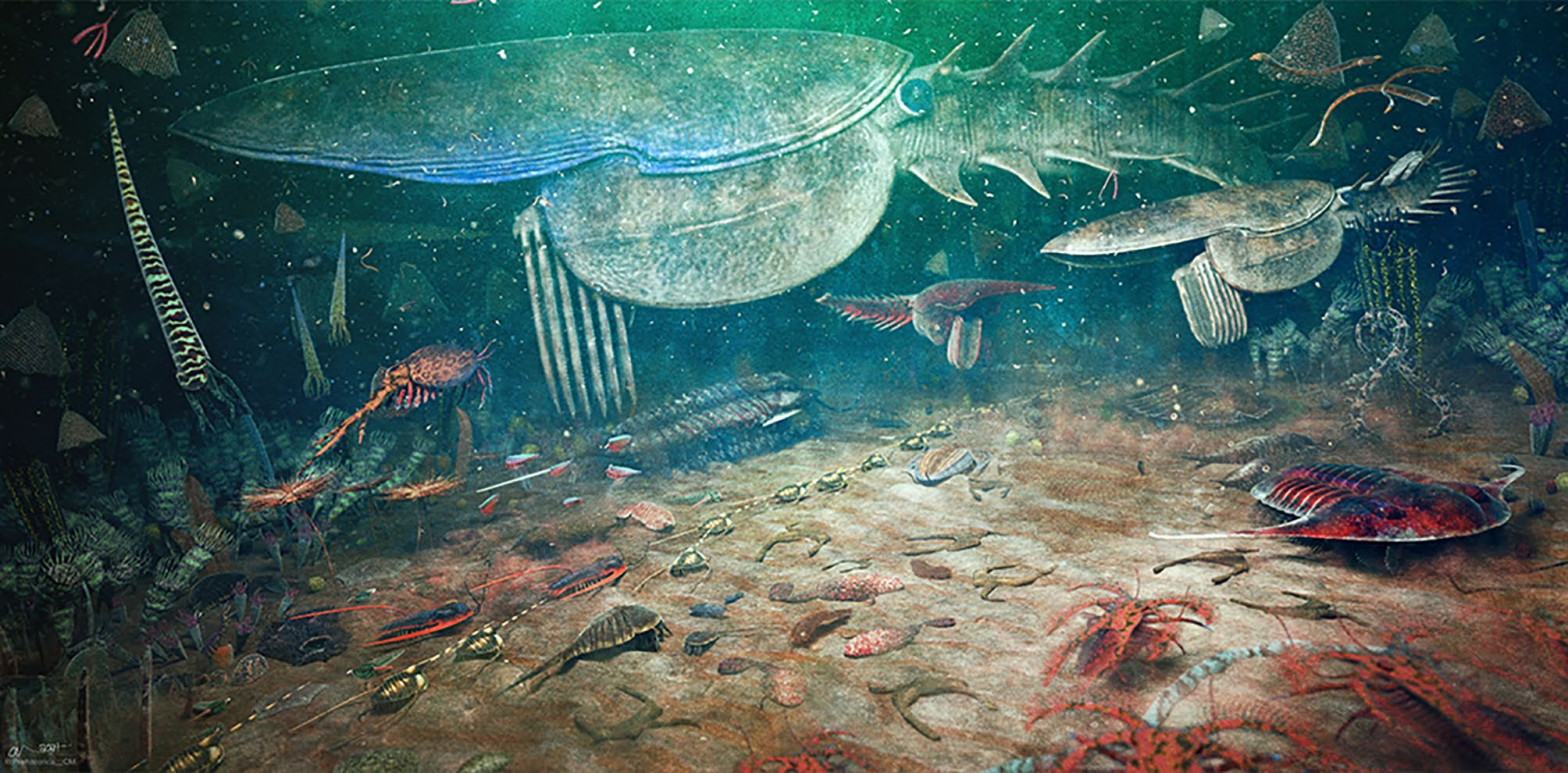
Artistic Reconstruction of the Fezouata Biota by Christian McCall (Prehistorica Art). An annotated figure is provided in the Supplementary Material.

This study explores preservation variations in the Early Ordovician Fezouata Biota. It shows that predators/scavengers are better preserved than suspension feeders and other feeding strategies such as grazers. No significant difference is observed between vagile and sessile organisms. The observed patterns of preservation result from complex taphonomic processes, and are also influenced by phylum-specific constraints. It is also shown that Tremadocian taxa in the Fezouata Biota are better preserved than Floian ones, which could reflect the gradual closure of the BST preservation window in the Zagora region of Morocco. In brief, there are many preservational variations in the Fezouata Biota (Fig. 4), similar to other Lagerstätten recording the rise of eumetazoans^56–58^. Acknowledging and understanding these preservational variations is key for coherently interpreting ancient snapshots of animal life on Earth.

## Supplementary Information

Supplementary Information.

## Data Availability

All the data needed to reproduce this paper are available in the main text and supplementary material.
